# Supplementary material for: Bacterial extracellular vesicles as recyclable nutrient reservoirs
Source: Nat Commun. 2026 Apr 6;17:4901. doi: 10.1038/s41467-026-71463-3 (PMC13230831; doi:10.1038/s41467-026-71463-3)
Supplement: Supplementary file 1 — Supplementary Information [file 41467_2026_71463_MOESM1_ESM.pdf]

SUPPLEMENTARY DATA

Bacterial extracellular vesicles as recyclable nutrient reservoirs

Astrid Laimer-Digruber[1,2#], Tanja V Edelbacher[1,3#§], Masoumeh Alinaghi[1], Mia S C Yu[3], Dapi Menglin Chiang[3,4,5], Benedikt Kirchner[3,5], Susanne I Wudy[6], Waltraud Tschulenk[7], Ingrid Walter[7], Stefan Kummer[8], Christina Ludwig[6], Jan Přibyl[2], Michael W Pfaffl[3], Monika Ehling-Schulz[1*]

[1]Institute of Microbiology, Centre of Pathobiology, Department of Pathobiology and Biomedical Sciences, University of Veterinary Medicine, Vienna, Austria

[2]CEITEC MU, Masaryk University, Brno, Czech Republic

[3]Division of Animal Physiology and Immunology, School of Life Sciences Weihenstephan, Technical University of Munich (TUM), Freising, Germany

[4]Department of Biomedicine, University of Basel, Basel, Switzerland

[5]Institute of Human Genetics, University Hospital, LMU Munich, Munich, Germany

[6]Bavarian Center for Biomolecular Mass Spectrometry (BayBioMS), TUM School of Life Sciences, Technical University of Munich (TUM), Freising, Germany

[7]Institute of Morphology, Centre of Pathobiology, Department of Pathobiology and Biomedical Sciences, University of Veterinary Medicine, Vienna, Austria

[8]VetCore Facility for Research, University of Veterinary Medicine, Vienna, Austria

[#]Contributed equally

[§]Current address: CDL Research, University Medical Center Utrecht, Utrecht, The Netherlands.

[*]Corresponding author, monika.ehling-schulz@vetmeduni.ac.at

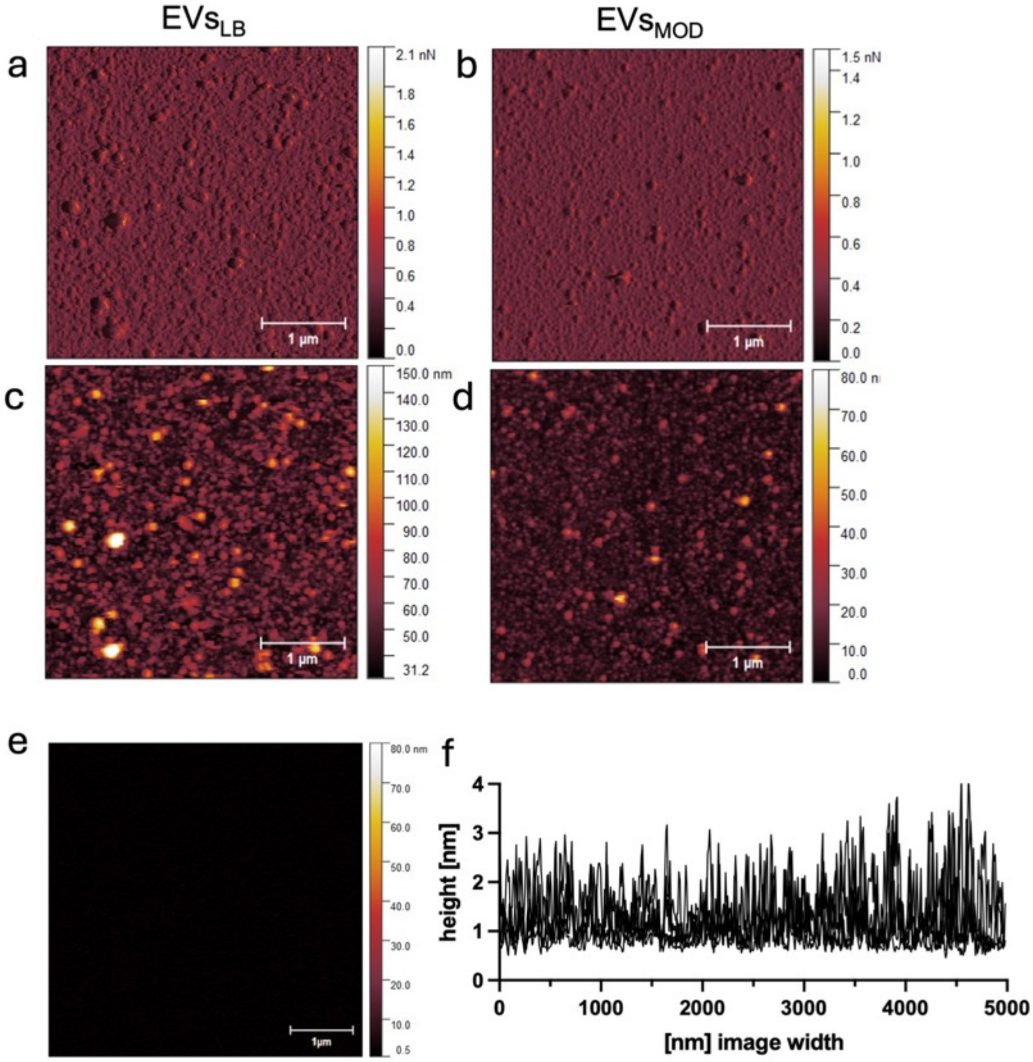

Supplementary Fig. 1: Atomic force microscopy (AFM) of EVs$_{LB}$ and EVs$_{MOD}$ shows vesicular structures. **a-d** AFM images of EVs$_{LB}$ or EVs$_{MOD}$ collected from *Bacillus cereus* cultivated in either LB or MOD for 9 h at 30°C, 120 rpm show the peak force error (**a, b**) or height profile (**c, d**). **e, f** Mock-incubated slides (PBS) were used as controls, showing the height profile (**e**) and five representative line profiles spanning the whole image (**f,** n=5 biological replicates). Source data are provided as Source Data file.

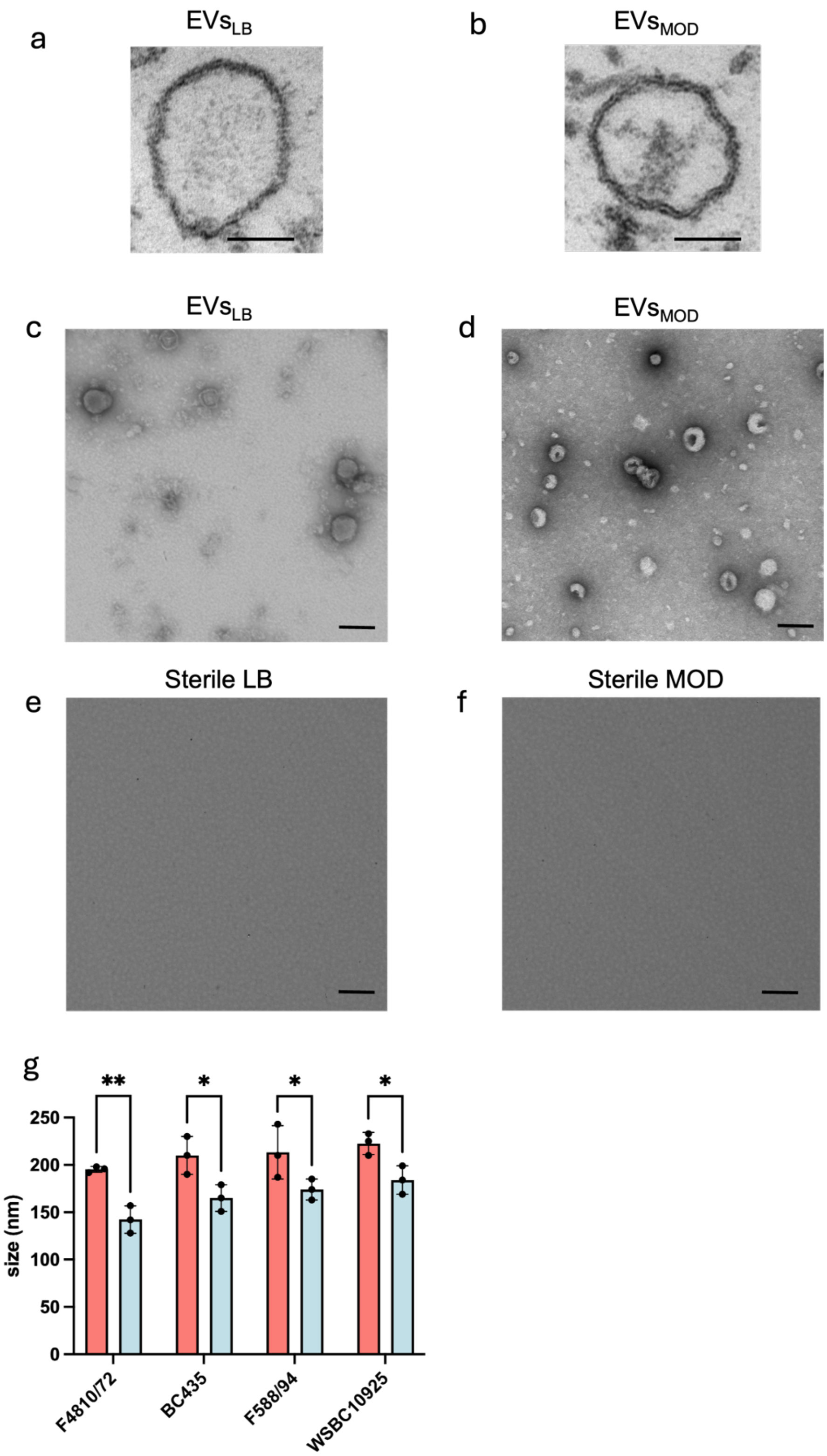

Supplementary Fig. 2: Transmission electron microscopy and NTA confirm characteristic EV morphology and size. **a-d** Transmission electron microscopy (TEM) was performed on EVs of *B. cereus* F4810/72 harvested after 9 h of bacterial cultivation in LB or MOD. **a, b** EVs were imaged by either preparing resin-embedded ultrathin sections or by **c, d** the drop-on-grid method. **e, f** In addition, sterile-media was also inspected by employing the drop-on-grid method utilising TEM. The scale bar in TEM images **(a-f)** indicates 100 nm. **g** Utilising NTA, EV sizes were further compared between F4810/72 and other *B. cereus* strains (n=3). Statistical differences were assessed using one-way ANOVA with Bonferroni's multiple comparison test. Source data are provided as Source Data file.

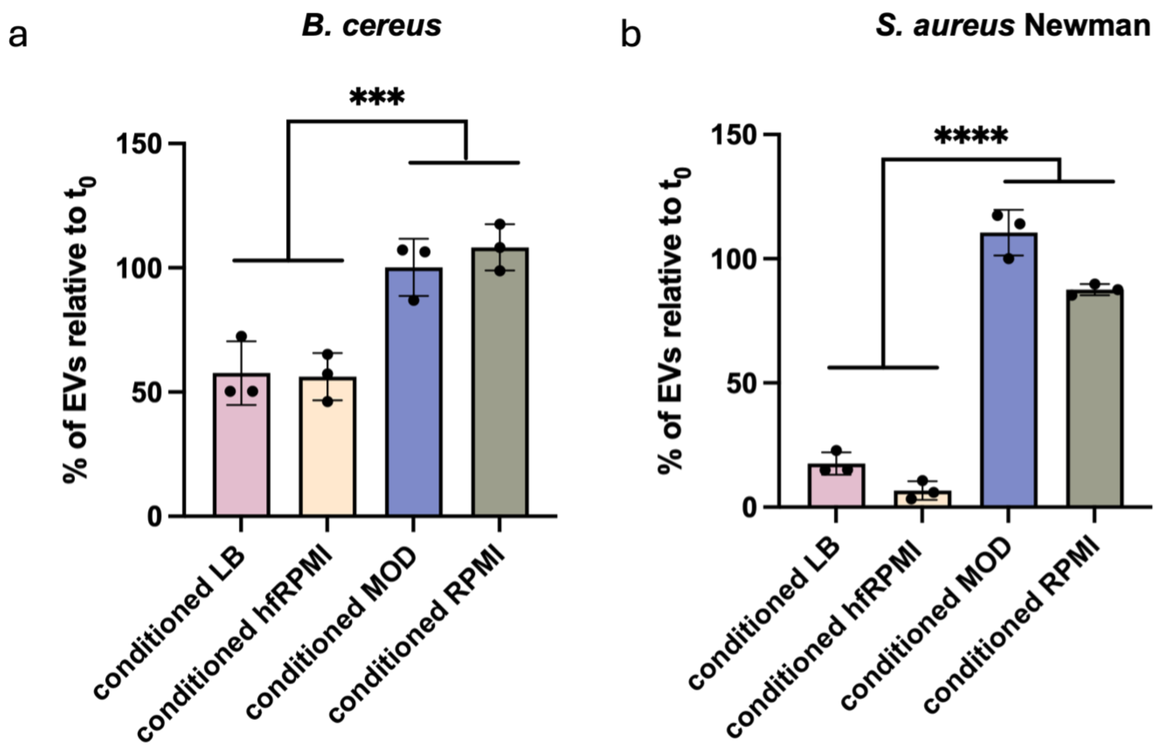

Supplementary Fig. 3: EV degradation is not species specific and occurs only in nutrient-complex media. EV degradation was measured in bacterial cell-free conditioned media (LB, host factor-enriched RPMI (hfRPMI), MOD, RPMI). *B. cereus* (**a**; n=3 biological replicates) or *Staphylococcus aureus* (**b**, n=3 biological replicates) were grown in the respective media at 30°C, 120 rpm. After 7 h, bacterial cells were removed, and EV counts were determined (t_0). Subsequently, this cell-free conditioned media containing EVs was further incubated for 4.5 h at 30 °C, 120 rpm to mimic bacterial culture conditions in the absence of bacterial cells. The relative number of EVs present after 4.5 h to t_0 was calculated. Statistical differences were assessed using two-way ANOVA with Bonferroni's multiple comparison test. ***$p < 0.001$, ****$p < 0.0001$. Source data are provided as Source Data file.

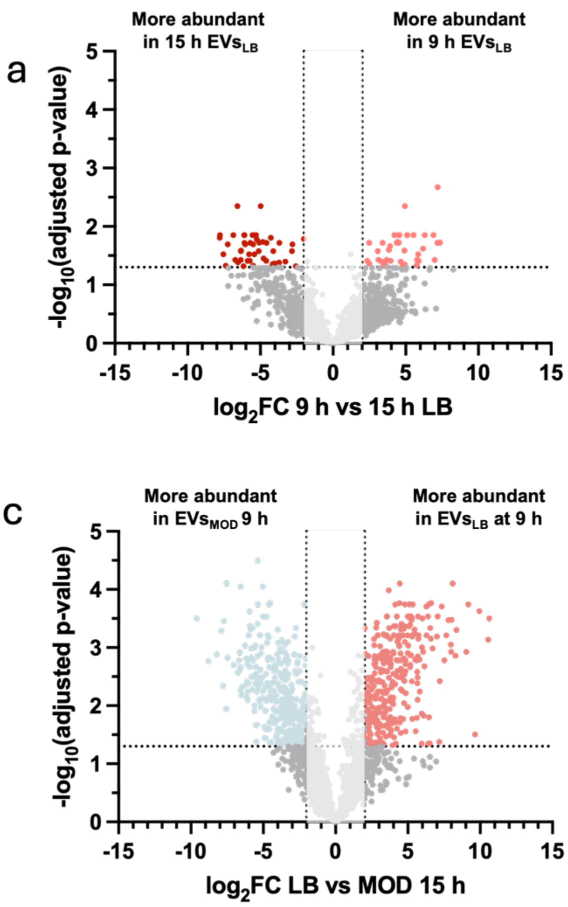

Supplementary Fig. 4: The proteomes of EVs are highly influenced by culture condition and culture duration. **a-d** The EV proteome data were subjected to volcano plot analysis. As cut-offs for statistical significance, a log_2FC of 2, *and* an adjusted p-value of –log1.3 were used. For proteomic analysis, n=3 biological replicates were sampled for EVs_{LB} 15 h, n=4 biological replicates were used for all other samples (EVs_{LB} 9 h, EVs_{MOD} 9 h and EVs_{MOD} 15 h).

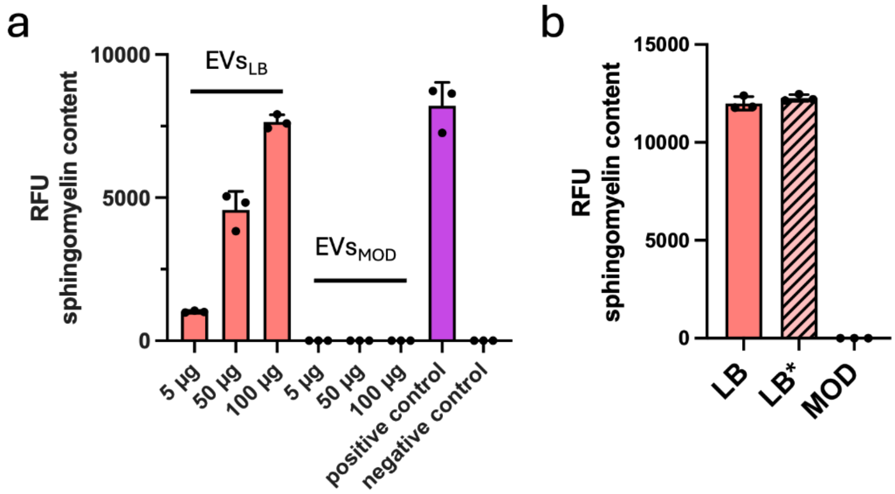

Supplementary Fig. 5: A targeted sphingomyelin assay confirms EV lipid bilayer characteristics. A fluorometric sphingomyelin kit was used to quantify sphingomyelins in *B. cereus* F4810/72 EVs and media. **a** Only EVs$_{LB}$ contained sphingomyelin but not EVs$_{MOD}$ (n=3 biological replicates). **b** The sphingomyelin amount was also quantified in sterile LB, sterile ultracentrifuged LB (LB*) and in sterile MOD. No significance difference was observed between LB and ultracentrifuged LB (n=3 independent experiments). No sphingomyelin was detected in sterile MOD media. Source data are provided as Source Data file.

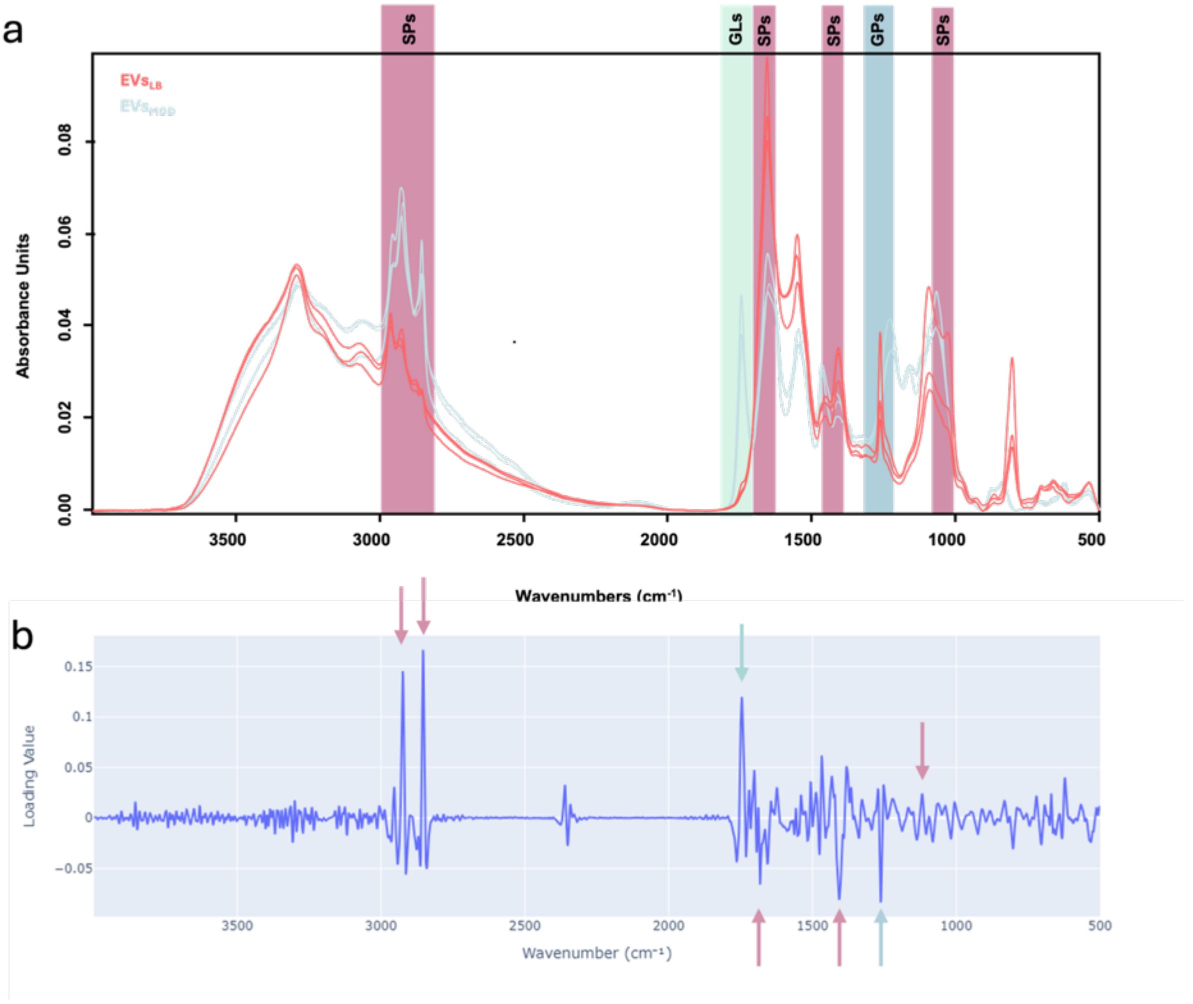

Supplementary Fig. 6: Impact of culture media on the EV lipidome revealed by Fourier-transform infrared spectroscopy (FTIR). Preprocessed FTIR spectra were used for chemometric analysis to gain insights into changes of EV lipid building blocks. **a** FTIR spectral fingerprints were generated from EVs_{LB} and EVs_{MOD} isolated from bacteria cultivated for 9 h at 30°C, 120rpm. Spectral data were normalised and baseline corrected. Coloured regions show regions accounting for sphingolipids, glycerolipids and glycerophospholipids (n=3 biological replicates). **b** PC1 loading plot obtained from PCA (Fig. 2c) shows multivariate differences between EVs_{LB} and EVs_{MOD}. Peaks highlighted by arrows indicate important wavenumbers that account for spectral differences in the lipid composition of EVs.

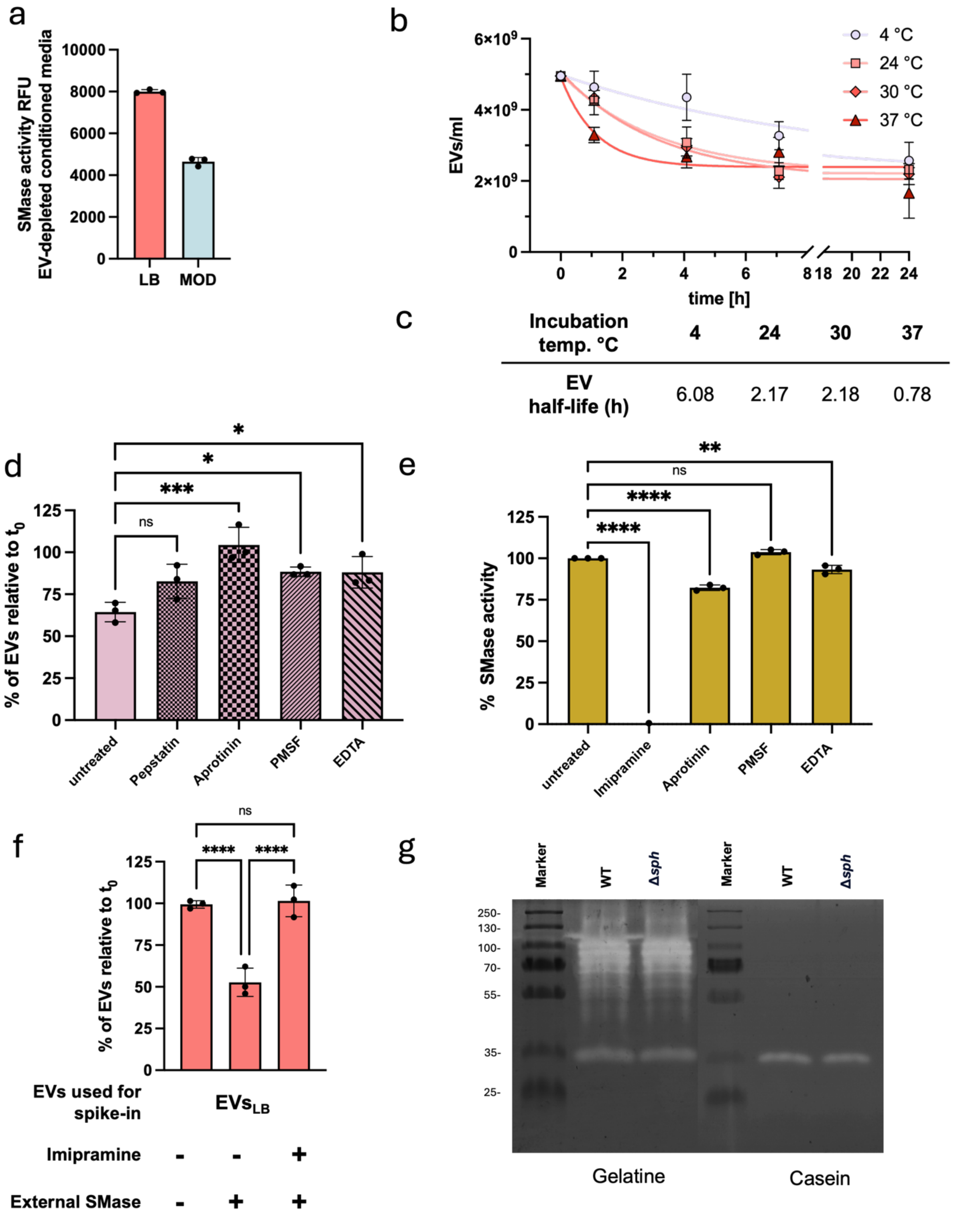

Supplementary Fig. 7: EV-degradation is enzyme-mediated and temperature dependent. a - d, h Cell-free conditioned LB media containing endogenous bacterial EVs was prepared by growing bacterial cultures for 7 h and removing bacterial cells by centrifugation and sterile filtration. **a** To determine SMase production of *B. cereus* F4810/72, the conditioned media were EV-depleted by ultracentrifugation (n=3). SMase production was tested by Amplex® Red Sphingomyelinase Assay Kit. **b** The effect of temperature on EV degradation was investigated by dividing cell-free conditioned EV-containing media into subsamples and exposing these samples to different temperatures (4 °C, 24 °C,

30 °C, 37 °C; (n=3 for each condition)). EV amounts were assessed at the times indicated. **c** The effect of temperature on EV degradation was quantified using one-phase decay curves, showing the respective EV half-life. **d** To test whether pharmacological substances can influence EV degradation, different protease inhibitors were added to the cell-free conditioned EV-containing media. The EV amounts were determined at t_0 and 4.5 h after the start of the experiment (n=3). **e** To assess the SMase-inhibitory potential of Imipramine and the protease inhibitors Aprotinin, PMSF, and EDTA, these compounds were added to the SMase activity assay. **f** To confirm the specificity of Imipramine-mediated SMase inhibition in the experimental setting (Fig. 4b), exogenous SMase was added to EVs_{LB}-spiked LB medium, resulting in EV degradation. Addition of Imipramine prevented SMase-mediated EV degradation. **d, e, f** Statistical significance was determined by one-way ANOVA with Bonferroni's multiple comparison test. *$p < 0.05$, **$p < 0.01$, ***$p < 0.001$, ****$p < 0.0001$. **g** To assess the proteolytic potential of bacterial cultures, zymography using either gelatine or casein as a substrate was conducted using EV-depleted, cell-free conditioned LB in which WT or Δ*sph* were grown. Source data are provided as Source Data file.

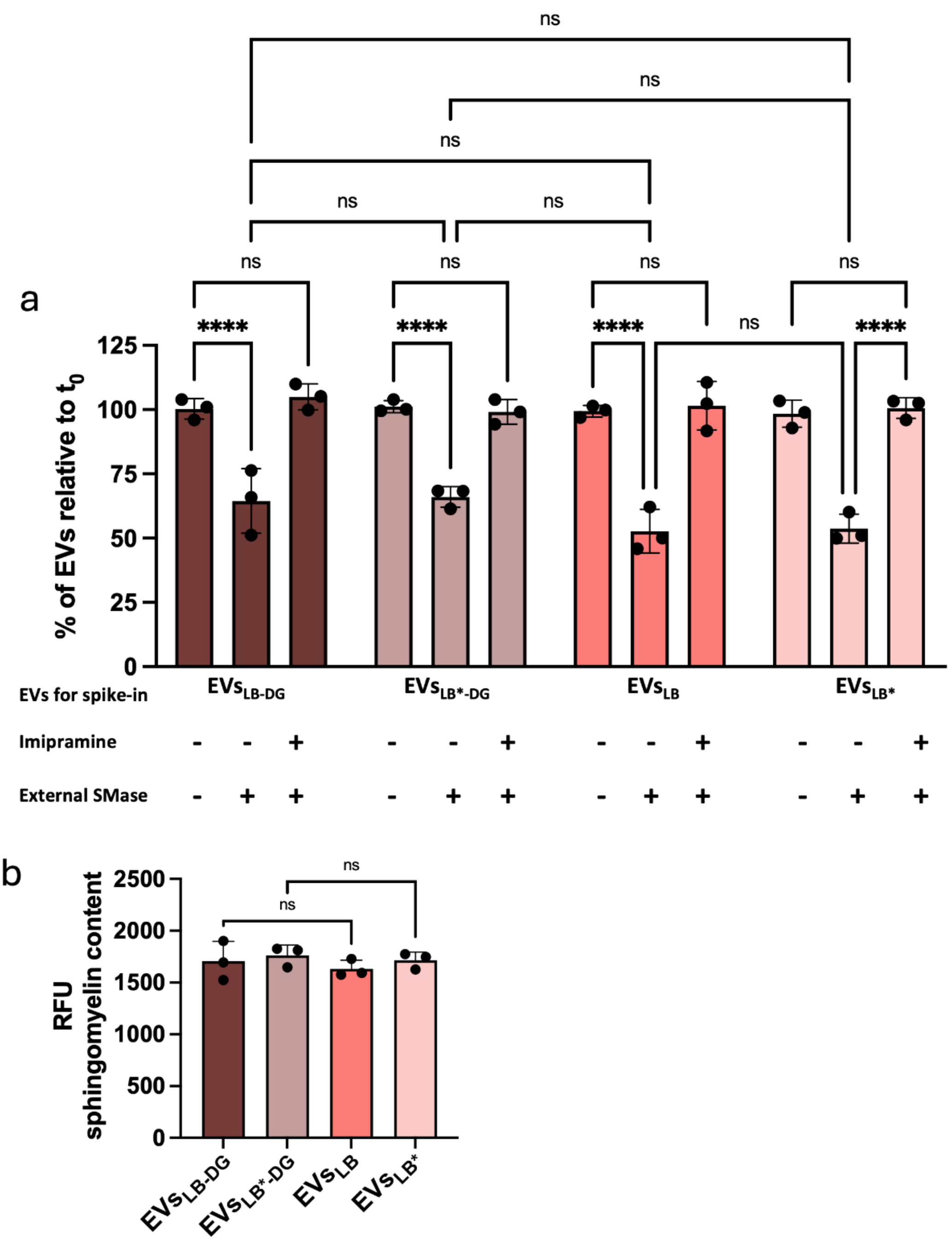

Supplementary Fig. 8: ***SMase-mediated EV degradation experiments of density gradient-purified EVs parallel results from EVs$_{LB}$.*** **a** To exclude potential effects of co-purified components on EV degradation, EVs derived from bacteria grown in LB (EVs$_{LB}$) were further purified by density gradient ultracentrifugation and used for spike-in experiments (EVs$_{LB-DG}$). In addition, density gradient–purified EVs obtained from bacteria grown in ultracentrifuged LB (LB*) were included (EVs$_{LB*-DG}$). For reasons of comparison, EVs$_{LB}$ (original data is shown in Supplementary Fig. 7f) and EVs$_{LB*}$ were included in this experiment. EVs$_{LB-DG}$, EVs$_{LB*-DG}$, EVs$_{LB}$, EVs$_{LB*}$ were spiked into sterile LB, sterile LB supplemented with external SMase, or sterile LB containing both external SMase and imipramine. EV degradation was observed only in the presence of exogenous SMase, whereas EV degradation was inhibited upon addition of imipramine. **b** A quantitative sphingomyelin assay using EVs$_{LB-DG}$, EVs$_{LB*-DG}$, EVs$_{LB}$ and EVs$_{LB*}$ showed similar sphingomyelin levels. **a, b** Data presented reflect mean ± SD of n=3 biological replicates. Statistical significance was determined by one-way ANOVA with Bonferroni's multiple comparison test.****$p < 0.0001$; ns: not significant. Source data are provided as Source Data file.

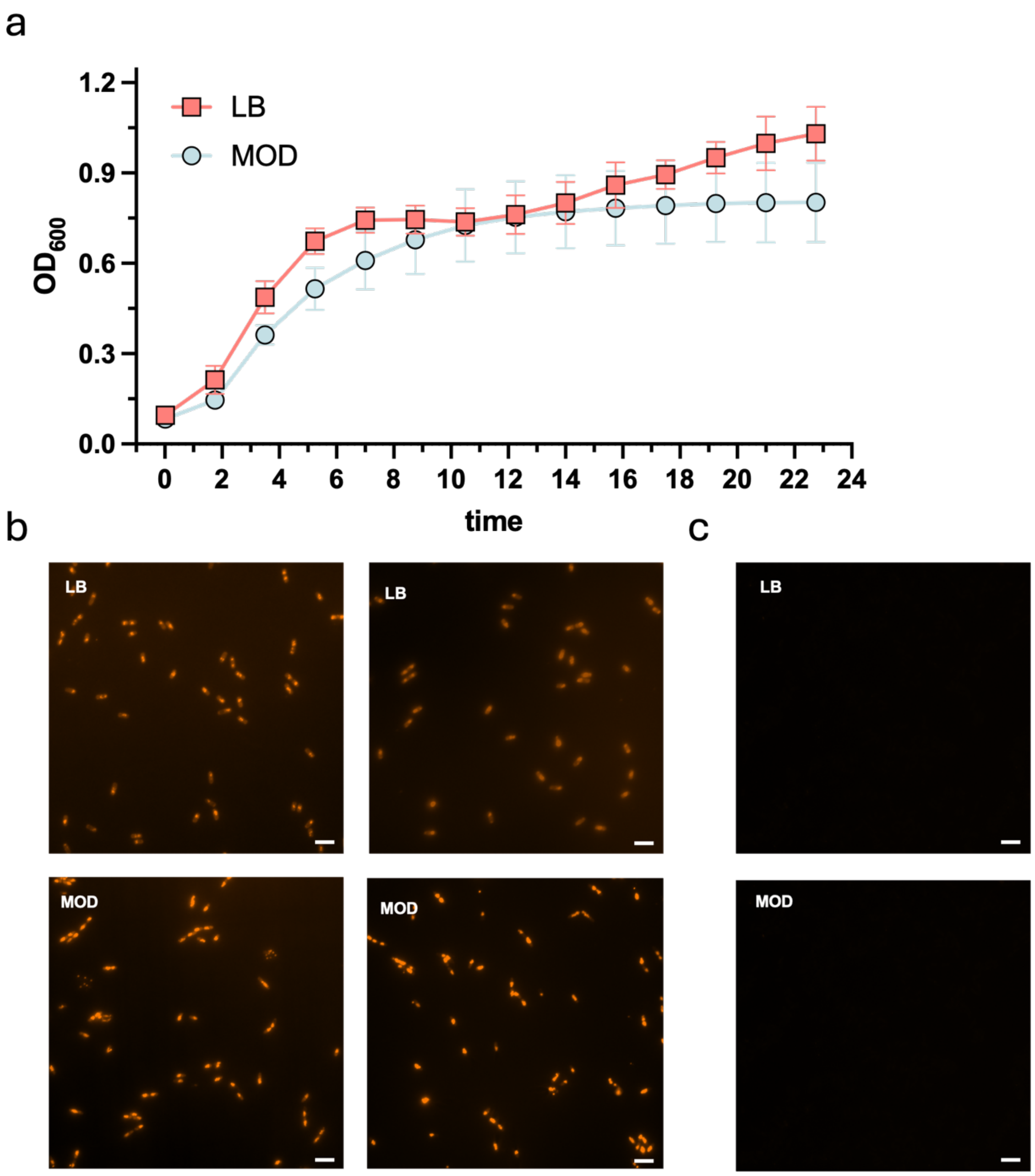

Supplementary Fig. 9: *Additional profiling of the bacterial growth and the redox state of bacterial cells.* **a** *B. cereus* growth in LB and MOD was monitored using an automated bacterial growth recorder. The growth curves shown provide the underlying data for the figures presented in Fig. 5a, b. **b** The fluorogenic ROS sensor CellROX™ was used to detect oxidative stress in bacteria grown in LB or MOD. **b** Representative image sections of additional biological replicates are shown, in addition to the representative images depicted in the main figure 5 (Fig. 5f). **c** To account for bacterial autofluorescence, a mock staining was performed (545/565 nm) and is shown for LB and MOD cultured bacteria. Source data are provided as Source Data file. Scale bar indicates 10 µm.
