# Supplementary material for: Bacterial extracellular vesicles as recyclable nutrient reservoirs
Source: Nat Commun. 2026 Apr 6;17:4901. doi: 10.1038/s41467-026-71463-3 (PMC13230831; doi:10.1038/s41467-026-71463-3)
Supplement: Supplementary file 2 — Description of Additional Supplementary Files [file 41467_2026_71463_MOESM2_ESM.pdf]

Description of Additional Supplementary Files:

Supplementary Data 1: Qualitative analysis of proteins present in EVs_{LB} and EVs_{MOD}

Supplementary Data 2: Differentially regulated proteins used for volcano plot

Supplementary Data 3: Lipid species identified in the respective EV lipidome

Supplementary Data 4: Stress-associated proteins identified in the respective *B. cereus* proteome of EVs_{LB} and EVs_{MOD}.

Supplementary Data 5: Identified RNA biotypes within the EV transcriptome

Supplementary Data 6: Composition of laboratory cultivation media
