# Supplementary material for: Bacterial extracellular vesicles as recyclable nutrient reservoirs
Source: Nat Commun. 2026 Apr 6;17:4901. doi: 10.1038/s41467-026-71463-3 (PMC13230831; doi:10.1038/s41467-026-71463-3)
Supplement: Supplementary file 9 — Reporting Summary [file 41467_2026_71463_MOESM9_ESM.pdf]

nature portfolio

Corresponding author(s): Monika Ehling-Schulz

Last updated by author(s): Feb 16, 2026

Reporting Summary

Nature Portfolio wishes to improve the reproducibility of the work that we publish. This form provides structure for consistency and transparency in reporting. For further information on Nature Portfolio policies, see our Editorial Policies and the Editorial Policy Checklist.

Statistics

For all statistical analyses, confirm that the following items are present in the figure legend, table legend, main text, or Methods section.

n/a	Confirmed	
☐	☒	The exact sample size (*n*) for each experimental group/condition, given as a discrete number and unit of measurement
☐	☒	A statement on whether measurements were taken from distinct samples or whether the same sample was measured repeatedly
☐	☒	The statistical test(s) used AND whether they are one- or two-sided<br>*Only common tests should be described solely by name; describe more complex techniques in the Methods section.*
☒	☐	A description of all covariates tested
☐	☒	A description of any assumptions or corrections, such as tests of normality and adjustment for multiple comparisons
☐	☒	A full description of the statistical parameters including central tendency (e.g. means) or other basic estimates (e.g. regression coefficient) AND variation (e.g. standard deviation) or associated estimates of uncertainty (e.g. confidence intervals)
☐	☒	For null hypothesis testing, the test statistic (e.g. *F*, *t*, *r*) with confidence intervals, effect sizes, degrees of freedom and *P* value noted<br>*Give P values as exact values whenever suitable.*
☒	☐	For Bayesian analysis, information on the choice of priors and Markov chain Monte Carlo settings
☒	☐	For hierarchical and complex designs, identification of the appropriate level for tests and full reporting of outcomes
☒	☐	Estimates of effect sizes (e.g. Cohen's *d*, Pearson's *r*), indicating how they were calculated

Our web collection on statistics for biologists contains articles on many of the points above.

Software and code

Policy information about availability of computer code

Data collection	Data was organized and collected in Microsoft Excel (Version 2502) Build 16.0.18526.20546), Microsoft PowerPoint (Version 2502 Build 16.0.18526.20546) and Graph Pad Prism (Version 10.4.2 (534)) for Windows and Mac. Nanoparticle tracking analysis (NTA) data was collected using a PMX-230 TWIN Laser ZetaView with the ZetaView 8.04.02 software.
Data analysis	For AFM processing and analysis: Bruker NanoScope Analysis (v3.0 64bit) and Gwyddion (v 2.67 64bit) were used. For FTIR data analysis Python (version 3.11.0) using the following libraries: pandas (version 2.2.3), numpy (version 2.1.3) , matplotlib (version 3.8.4), scipy (version 1.15.1), and scikit-learn (version 1.6.1) were used in addition to OPUS 8.0. For initial proteomic data analysis MaxQuant (Version 1.6.3.4) and Perseus (Version v2.0.10.0) was used, after quantile-normalization datasets were imported into an R script for further analysis, the R script has been deposited to the following platforms: https://zenodo.org/records/16752325; DOI: 10.5281/zenodo.16752325.<br>Python (version 3.11.0) using the following versions of libraries: pandas (2.3.3), numpy (2.1.3), plotly (6.0.0), seaborn (0.13.2), scikit-learn (1.6.1), goatools (1.4.12) were used. For transciptomic analysis following softwares were used: cutadapt (v4.4), FastQC (v0.12.1), bowtie (v1.3.1), HTSeq (v0.11.1).

For manuscripts utilizing custom algorithms or software that are central to the research but not yet described in published literature, software must be made available to editors and reviewers. We strongly encourage code deposition in a community repository (e.g. GitHub). See the Nature Portfolio guidelines for submitting code & software for further information.

Data

Policy information about availability of data

All manuscripts must include a data availability statement. This statement should provide the following information, where applicable:

- Accession codes, unique identifiers, or web links for publicly available datasets
- A description of any restrictions on data availability
- For clinical datasets or third party data, please ensure that the statement adheres to our policy

The data generated in this study are provided in the Source Data file. The mass spectrometric raw files as well as the MaxQuant output files concerning proteomics have been deposited to the ProteomeXchange Consortium via the PRIDE repository and can be accessed using the identifier PXD065751 (https://proteomecentral.proteomexchange.org/cgi/GetDataset?ID=PXD065751), reviewer account username: reviewer_pxd065751@ebi.ac.uk, password: 2kGLi3g78kdH). The mass spectrometric raw files as well as the output files concerning lipidomics have been deposited to the MetaboLights repository with the study identifier MTBLS13260. The transcriptomic data is deposited at European Nucleotide Archive (ENA Accession Number: PRJEB94405) and is available during the review process.

Research involving human participants, their data, or biological material

Policy information about studies with human participants or human data. See also policy information about sex, gender (identity/presentation), and sexual orientation and race, ethnicity and racism.

Reporting on sex and gender	No human research participants were involved in this study.
Reporting on race, ethnicity, or other socially relevant groupings	No human research participants were involved in this study.
Population characteristics	No human research participants were involved in this study.
Recruitment	No human research participants were involved in this study.
Ethics oversight	No human research participants were involved in this study.

Note that full information on the approval of the study protocol must also be provided in the manuscript.

Field-specific reporting

Please select the one below that is the best fit for your research. If you are not sure, read the appropriate sections before making your selection.

☒ Life sciences ☐ Behavioural & social sciences ☐ Ecological, evolutionary & environmental sciences

For a reference copy of the document with all sections, see nature.com/documents/nr-reporting-summary-flat.pdf

Life sciences study design

All studies must disclose on these points even when the disclosure is negative.

Sample size	Sample size estimations were not performed, but a minimum of three independent replicates was performed in order to have sufficient replicates for statistical tests, which has been proven to be sufficient for bacteria work based on the lab's experience.
Data exclusions	No data was excluded in this manuscript.
Replication	Experiments were repeated multiple times independently from each other and could be repeated across different experimentators.
Randomization	All findings were independent of the order of experimental replicates.
Blinding	Blinding was not relevant to this study.

Reporting for specific materials, systems and methods

We require information from authors about some types of materials, experimental systems and methods used in many studies. Here, indicate whether each material, system or method listed is relevant to your study. If you are not sure if a list item applies to your research, read the appropriate section before selecting a response.

Materials & experimental systems

n/a	Involved in the study
☒	☐ Antibodies
☐	☒ Eukaryotic cell lines
☒	☐ Palaeontology and archaeology
☒	☐ Animals and other organisms
☒	☐ Clinical data
☒	☐ Dual use research of concern
☒	☐ Plants

Methods

n/a	Involved in the study
☒	☐ ChIP-seq
☒	☐ Flow cytometry
☒	☐ MRI-based neuroimaging

Eukaryotic cell lines

Policy information about cell lines and Sex and Gender in Research

Cell line source(s)	Caco-2 cells were obtained from ATCC, which were originally isolated from a male patient with colorectal adenocarcinoma.
Authentication	None of the cell lines were authenticated.
Mycoplasma contamination	Caco-2 cells are regularly tested for mycoplasma contamination.
Commonly misidentified lines (See ICLAC register)	na

Plants

Seed stocks	na
Novel plant genotypes	na
Authentication	na
